# “That’s why we’re speaking up today”: exploring barriers to overdose fatality prevention in Indianapolis’ Black community with semi-structured interviews

**DOI:** 10.1186/s12954-023-00894-8

**Published:** 2023-10-27

**Authors:** Dong-Chul Seo, Naomi Satterfield, Leonardo Alba-Lopez, Shin Hyung Lee, Charlotte Crabtree, Nicki Cochran

**Affiliations:** 1 School of Public Health, Indiana University Bloomington, IN, 1025 E 7th St., Bloomington, IN 47405 USA; 2 School of Education, Indiana University Bloomington, IN, 201 N Rose Ave., Bloomington, IN 47405 USA; 3 Overdose Lifeline, Inc., Indianapolis, IN, 1100 W 42Nd St., Suite 385, Indianapolis, IN 46208 USA

**Keywords:** Opioid overdose fatalities, Racism, Naloxone, Harm reduction, Stigma, Fear, Mistrust, First responders

## Abstract

**Background:**

Opioid overdose deaths are of great concern to public health, with over one million lives lost since 1999. While many efforts have been made to mitigate these, Black communities continue to experience a greater burden of fatalities than their white counterparts. This study aims to explore why by working with Black community members in Indianapolis through semi-structured interviews.

**Methods:**

Semi-structured one-on-one in-depth interviews were conducted in spring and summer of 2023 with Black residents (*N* = 23) of zip codes 46202, 46205, 46208, and 46218 in Indianapolis. Ten interview questions were used to facilitate conversations about opioid overdoses, recovery, fatality prevention tools such as calling 911 and naloxone, law enforcement, and racism. Data were analyzed using grounded theory and thematic analysis.

**Results:**

Interviews revealed access barriers and intervention opportunities. Racism was present in both. Mental access barriers such as stigma, fear, and mistrust contributed to practical barriers such as knowledge of how to administer naloxone. Racism exacerbated mental barriers by adding the risk of race-based mistreatment to consequences related to association with substance use. Participants discussed the double stigma of substance use and being Black, fear of being searched in law enforcement encounters and what would happen if law enforcement found naloxone on them, and mistrust of law enforcement and institutions that provide medical intervention. Participants had favorable views of interventions that incorporated mutual aid and discussed ideas for future interventions that included this framework.

**Conclusions:**

Racism exacerbates Blacks' mental access barriers (i.e., help-seeking barriers), which, in turn, contribute to practical barriers, such as calling 911 and administering naloxone. Information and resources coming from people within marginalized communities tend to be trusted. Leveraging inter-community relationships may increase engagement in opioid overdose fatality prevention. Interventions and resources directed toward addressing opioid overdose fatalities in Black communities should use mutual aid frameworks to increase the utilization of the tools they provide.

**Supplementary Information:**

The online version contains supplementary material available at 10.1186/s12954-023-00894-8.

## Background

Opioid-involved overdoses pose a substantial concern for public health. Over the period since 1999, more than one million have lost their lives due to drug overdoses [1]. In 2021, opioids caused more than 75% of all drug overdose fatalities [2]. Reflecting the same nationwide public health concerns, the state of Indiana experienced a similar pattern. In 2021, a total of 2,811 individuals succumbed to drug overdoses in the state, of which 2,321 deaths were linked to opioids [3]. Further, the opioid crisis in Indiana poses a distinctive challenge due to its impact on Black communities [4]. This challenge is exacerbated by the fact that Black individuals are experiencing greater number of opioid-involved overdose deaths compared to their white counterparts [4].

In response to the epidemic, major efforts have been made to mitigate opioid overdose fatalities, including allocating fundings for the implementation of naloxone-based interventions and enacting laws such as Good Samaritan laws [5]. Despite the efforts, the numbers of opioid overdose fatalities continue to increase, indicating a need to closely examine factors associated with the rise. One possible rationale behind this ongoing increase could be the influence of mental barriers, including stigma, fear, mistrust, and hostility which are connected to structural racism [6–11]. The presence of those mental barriers toward law enforcement within the population of people who use drugs (PWUDs) and among residents in marginalized communities can exacerbate the problems related to drug use for several reasons [7–11]. First, individuals who fear or mistrust law enforcement exhibit greater levels of reluctance to make 911 calls in overdose situations [7]. Second, the stigma and hostility surrounding drug use deter PWUDs from seeking medical assistance due to concerns about potential arrest or criminal charges [8–10]. Lastly, the avoidance of seeking medical assistance has the potential to worsen social disparities by hindering individuals’ access to timely and proper healthcare services. This, in turn, can lead to worsened marginalization, including poor health outcomes and an increase in social stigma [11]. In addition, these barriers could potentially influence aspects of health determinants, such as quality of healthcare, availability of community education, and the presence of social support systems, particularly within communities with large Black populations. Specifically, the inadequate fundamental infrastructure including limited access to addiction and mental health services might be exacerbating this public health issue [12].

Mutual aid is a multifaceted approach that encompasses peer support, empowerment, holistic outlook as well as cultural relevance [13]. Mutual aid has been a longstanding practice within communities of color and indigenous people who experience crises [13]. The findings of a recent study suggest that mutual aid can be a beneficial approach for helping individuals who are opioid users [14]. Given that existing methods such as naloxone-based interventions are not yielding desirable outcomes such as reduction of opioid overdose fatalities, mutual aid can be an appealing alternative that should be considered. Nevertheless, the current evidence regarding the effect of mutual aid in preventing opioid overdose among Black people in Indiana is limited.

The present research study aims to explore 1: how racism intersects with opioid overdose prevention efforts and 2: the alternative approaches for preventing overdose fatalities in Black communities in Indianapolis. For that purpose, we partnered with community members in the focus area, including the interviewer whose lived experience made her a valuable gatekeeper. Our aim was to increase trust with participants and provide a research environment where they felt able to share honestly [15]. Given the history of marginalization in the study community, we employed thematic analysis and grounded theory in our analysis to “[illuminate] the situations of people denied a public voice” [16].

## Methods

This study was conducted as part of a larger study, Multi-Sector and Multi-Level Community-Driven Approaches to Remove Structural Racism and Overdose Deaths in Black Indianapolis Communities (MACRO-B). The aims of this study were to explore how racism interacts with fatality reduction behaviors following an opioid overdose, such as calling 911 and administering naloxone, and to identify alternative approaches to preventing fatalities. As this study sought to explore the experiences of individuals with similar characteristics, homogenous sampling [17] was applied as well as snowball sampling to saturate the data [18], resulting in a blended approach. Participants were contacted via email, required to be Black residents over 18 years old who live in four specific zip codes of the Indianapolis area (46202–46205–46208–46218), and were compensated with a $50 gift card. Geolocation was relevant for the study because those areas are considered inner city, and have higher proportions of Black residents than other areas in Indiana. In 2021, the median incomes for the four zip codes were $54,972, $56,563, $41,076, and $28,758, respectively, while the proportions of residents living below 200% of the federal poverty level (FPL) were 36.6%, 32.7%, 42.7%, and 58.6% [19–22]. In addition, residents in these four zip codes experience some of the highest rates of overdose events and deaths in Indiana [23].

The MACRO-B Coalition is composed of various grassroots community partners, representatives of law enforcement, local emergency medical services, local fire department, decision-makers at local and state governments, local- and state-level legislators, and Black community residents who are overdose survivors or family members of fatal overdoses. Twenty-three semi-structured one-on-one interviews were conducted by a member of the study population, a Black woman with lived experience in substance use who was also a member of the Coalition. The interviewer, Charlotte, was part of the Overdose Lifeline team who, in 2020, found that Black Indianapolis residents were not being reached with naloxone. Her involvement in the grassroots effort to learn why, her experience in the Civil Rights movement, her residency in the study area, and her experience as a person in recovery from a substance use disorder made her an ideal interviewer. She was provided brief training on interviewing for qualitative research prior to data collection. She was also given instruction on getting informed consent and maintaining participant anonymity. We developed ten open-ended questions about participants’ experiences with opioid overdoses and first responders, knowledge of naloxone and barriers to opioid overdose fatality prevention such as community conditions, policies, and practices. Those question items were reviewed and edited by multiple community members and then pilot-tested with three Black community members. The nature of the interviews allowed the interviewer to use probes which served to have the interviewees clarify ideas or expand on responses. The interviews were audio recorded and transcribed verbatim using Temi by members of the Coalition. Data were de-identified, organized systematically after saturation [18] and stored into a matrix in a Microsoft Excel document to facilitate the creation of a codebook (see Additional File 1).

We used thematic analysis [25] and grounded theory [16] approaches to explore the barriers to opioid overdose fatality prevention as described and experienced by the participants. A systematic coding procedure—open, axial, and selective—[26] was followed by three members of the research team. This process allowed us to interact with the data sets, select salient excerpts, and analyze them constantly to generate categories and subcategories, facilitating the creation of a codebook. As data were collected and analyzed concurrently, we met once a week for two months to seek data that could challenge our expectations and emerging findings. We read and analyzed data separately, compared for duplication to avoid overlapping codes and integrated the refined codes into the codebook. We formed the themes by analyzing the major concepts found in the codebook and using QSR NVivo qualitative analysis software version 14.23.0. The software package aided our analysis process and allowed us to classify, sort, and arrange several pieces of information; and examine relationships in the data.

## Results

We found that interview excerpts could be separated into two major themes: access barriers and intervention opportunities, and that racism was ubiquitous throughout both. We focused on behaviors related to opioid overdose fatality prevention specifically. Narrations about substance use treatment or prevention are outside of the scope of this paper. Access barriers describe reasons why the study community may not engage in lifesaving behaviors, such as administering naloxone or calling 911, during an overdose event (see Additional File 2 for detailed quotes). While the race of the interviewer and participants and the interview questions suggest racism is implicit in most of the conversations, we chose to feature excerpts where race is explicit to show how it impacts access barriers. Attending to race specifically may reveal reasons for the disparate burden of overdose fatalities in the Black community. Intervention opportunities describe experiences participants report as helpful during an overdose event or when in need of substance use resources, or ideas they have to improve access to lifesaving substance use resources and behaviors (see Additional File 3 for detailed quotes).

### Access barriers

Interview participants frequently discussed two types of access barriers to engaging in lifesaving behaviors following an opioid overdose: practical barriers—factors that prevent the actions of administering naloxone or calling 911—and mental barriers—mindsets which drive the perception that administering naloxone and utilizing first responders are not appropriate behaviors for the individual. Consider both practical and mental barriers support a conceptualization of access that incorporates consumer beliefs like acceptability and trust along with supply factors like proximity and affordability [27].

#### Practical barriers

Practical barriers were reflected in physical and knowledge-related comments in which interviewees described availability of resources in their communities. Discussions about low knowledge of where to obtain and how to administer naloxone and the physical ability to go where resources are located were prevalent.

“The people who are out there in the madness, I'm not sure if someone's not bringing it to them, that they're getting it.”

“I don't even [want to] carry [naloxone because] I have no idea how to use none of that stuff.”

Some participants described low knowledge of legal rights when calling first responders following an overdose. For instance, participants discussed protective legislation such as “Aaron’s Law & Overdose Good Samaritan Law,” an Indiana law that protects individuals who administer naloxone in good faith when they call emergency medical services. Those who brought up legislation noted that both users and first responders lack appropriate legal knowledge.

“I'm running from you, the policeman. I'm, I'm gone. I don't know about no law. You don't even know nothing about the law. IMPD, you need to be educated about Aaron's Law because your initial thing is to arrest.”

#### Mental barriers

Mental barriers attended to perception and reasons why participants may have practical supply access to lifesaving behaviors and tools but not use them. They can be conceptualized as help-seeking barriers. Most observed barriers involved fear, mistrust, and stigma, which both stood alone and overlapped throughout the interviews. Some participants describe the fear that responding to an overdose may expose them to liability or cause harm.

“Instead of calling for help, they'll put a person outside...I do believe it's because people are intoxicated themselves at the moment. They're afraid of repercussions on themselves.”

“So many of [them] are afraid to use it because they're afraid that, ‘okay, I do this, and they don't come out of it maybe, and then they end up dying anyway. Maybe I did something wrong, maybe I was the one that killed them.’”

Participants also discussed low trust in emergency personnel and fear of interacting with police. This included hesitancy to call 911 and to carry naloxone, both of which were believed to increase risk of harassment from police.

“I would say there's a hesitancy because of the trust factor in our community. You know what I mean? We don't trust the police as it is already…When I was, uh, an employee at [social service], we would do a lot of outreach, stuff like that. And people were just not…they were scared because they, like, they didn't want to go to jail. They’re like, ‘We don't know nothing about [naloxone]. I'm not going to…’ they’re thinking like it's something wrong with it. Like, yeah, like ‘If the police catch me with this, maybe this will be an indication that I'm doing something.’”

Participants describe the belief that they will be stigmatized by first responders and others who may associate them with substance use if they have naloxone. Many expressed fear that substance use stigma held by first responders would make them vulnerable to mistreatment.

“Drugs do not have a color. Um, it affects all races. So, I believe, you know, um, it is because of, like I said, the stigma, um, them being possibly mistreated, you know? Whether it be police or it is, um, by individuals because people really don't have knowledge of understanding.”

“I've seen EMS treat people horribly…I've seen them call them junkies to their face. Like I've seen EMS behave horribly. Like, they have no type of stigma education whatsoever.”

“If they find [naloxone] on them, like they assume like, ‘Oh, if you got this then you may have some drugs somewhere. Let's go ahead and strip search her.’”

### Race

Race was discussed in relation to each of the above themes. The presence of race in interview responses and disparate overdose fatalities in the Black community led our team to attend specifically to how it emerged in discussions of barriers (Table 1). Many participants drew on examples from history where they observed what they could expect from first responders, interventions, and policies that have traditionally prioritized White populations. For instance, low proximity to resources such as substance use treatment centers and low knowledge about available resources within participants’ neighborhoods were linked to racism in Indianapolis’ resource distribution and the misconception that Black people do not use opioids.

“In the, uh, African American community, um, those individuals were forgotten, it appears. And, and a lot of those individuals don't know, uh, that there are resources out there.”

**“Interviewer:** Do you think that they, in the White community, they receive more education? **Subject**: I think so. I think they receive more education. They know what treatment sites they can go to. It just our community don't know. They just think there's nothing out there for you.”

**Table 1:** Participant quotes regarding race-related barriers to engaging in lifesaving behaviors following an opioid overdose

Theme	Quotation
Practical barriers: naloxone	The opioid or the opioid response in my community, uh, aims more so from my vantage point for White people. Um, it has been an epidemic. It became an epidemic when more White folks were in trouble with opioids. Um, for instance, the naloxone, you know, all these avenues that they have to, um, utilize before they go into, before, before they overdose in the African American community. I think the resources have been limited, but since it has become an epidemic for the Caucasian persuasion, um, I think it's, it's trickling down where it can be effective in the African American community
Practical barriers: naloxone	**Interviewer**: There are some legitimate claims that Black dominated communities do not receive equitable overdose education. **Subject**: Absolutely. **Interviewer**: And naloxone distribution as a White… **Subject**: As a matter of fact, in those communities what not, what, what education is there? Like as a, like that's just a research thing. If you just look. **Interviewer**: Mm-hmm. < affirmative > . **Subject**: It isn't there. There's, I mean, in my community it's a basically an everything desert
Practical barriers: naloxone	Well, this is the first time I ever see this in the Black community. What you guys are doing now, um, I go a lot to the [community organization] and also to [community organization]. Those are two things that are next to me. But, um, nobody never introduces or even had prevention, um, classes on drug use. Uh, you know, yes, sometimes I have to teach it and, and to the staff and train them on substance abuse, but we need to know this even deeper. Just don't hand us a, a package [of naloxone] and tell us “Have that in your car for an incident.” What I'm [going to] do if I have no clue how to use it, how to educate people?
Practical barriers: naloxone	The Black people don't understand how important it is to use…to know if you're [going to] use safely, check your stuff. Know who you’re getting it from, you know? Um, or just, you might just don't even know how to [administer naloxone]. Then you might need somebody to teach you how to [administer] it
Practical barriers: first responders	I think they didn't, they just don't care until something like, drastic in the news comes. They don't, you know, they're not caring, like actually caring to stop a person, like coming off of the street. “What you got?” you know, “Where you get it from?” you know, stuff like that. But if you're in a White neighborhood and you see some, a Black person coming, they're [going to] stop [them] and you know, check [them] and because they don't want you in their neighborhood buying drugs. But it's okay to buy drugs in a Black community. You know?
Practical barriers: first responders	Neighborhoods, um, they don't like coming to quote unquote “ghettos.” You know? They'd rather pull up and, you know, it's proportionally more, even though it's getting better, it's proportionally more Black people that are living [in] impoverished neighborhoods. So, um, yeah. So, less response, slower response rates into those areas specifically. Whereas I say on the other end, it's a Carmel overdose and they're there in two seconds
Practical barriers: first responders	**Interviewer**: Okay. So, but do you fear that if you had naloxone and you got pulled over for a ticket and the police saw the naloxone, do you think that they would harass you? **Subject**: Absolutely…I, I think that their history with working with our community is, uh, guilt first. Um, judge prosecutor executioner first, and then we'll find out that it's naloxone later
Practical barriers: first responders	A lot of times they don't, you know, because, uh, of who we are, you know, we have always been second fiddle. You know? We're not, uh, important, uh, to some of the, uh, emergency personnel, you know, that come, uh, to the scene of a crime or whatever. Uh, they're more apt to respond to their demographic, to people that look just like them
Mental barriers: stigma	**Subject**: Um, a White addicts get a little bit more sympathy than our Black, than their Black counterparts, so. Okay. Um, yeah. **Interviewer**: Okay. When you say, when you say they get more, more sympathy, you think people understand the disease in reference to them? **Subject**: It's more of a, “they are having a mental illness” for a White counterpart and it is more of a…where a Black person's more, “they were just lazy and they're just drug addicts.” Okay. So, you know, I've seen it with my own eyes, so
Mental barriers: stigma	My vantage point is that when individuals, again, of, of a certain ethnicity are stopped and they're perceived as, as violent or, uh, non-compliant
mental barriers: stigma	What I mean…unfortunately, you know, we, we still have that stigma of the police and it's just, it's the true stigma. I mean, it's true, you know, um, shoot first, ask questions later. And that's a scary thing, especially for young Black men, you know?
Mental barriers: stigma	**Interviewer**: Do you think race plays a part? **Subject**: Definitely. I think it has to do with, yeah…I think it has to do with where they’re being dispatched to. Like the areas, the communities. Cause when you, when you hear of a certain area, you know what type of people are there
Mental barriers: mistrust	You know, the Black community, that's med for one…that's medicine, real medicine from the cops giving it to [them]. The ambulance people are trying to give it to [them] and they don't even want it. Which is why I feel like one of the reasons…I feel like there's such a struggle with it is because they don't trust it
Mental barriers: mistrust	**Subject:**…the [affluent] communities, they're more friendly, they're more willing to talk to you. They're more willing to gain knowledge then our Black communities are. **Interviewer**: So you think we're, um, **Subject**: Stuck up. **Interviewer**: We're, we're not…you think we're stuck up? **Subject**: Okay. [We’re] stuck up. **Interviewer**: Okay. Why are we stuck up? What causes that? **Subject**: The way we've come up, our lifestyle, the things we've seen, the families we grew up with, us trying to build these walls from our childhood. Like, not knowing who to trust and who not to. It just makes us nonchalant and stuck up
Mental barriers: mistrust	So, yes. That's the only thing is, you know, as, as a race we've grown to be weary of stuff that's offered to us. It's always a catch. So, it is just about getting to the right people
Mental barriers: fear	**Interviewer**: What proportion of the residents in your community do you think would be, uh, fearful of carrying naloxone if they think the police might…? **Subject**: I think 80, like 80%. And I'm like you, that the Black community don't know, um, a lot and probably gets the information, you know, the, at the end of the stick
Mental barriers: fear	I don't believe that, um, that they’re so much [afraid] of being arrested, like for the possession of drugs. It's a bigger problem. And television and what's going on around the world, um, has affected how people look at the police. So probably everybody is fearing < laugh > , you know, for [their] life if they get stopped. [Because] it's so much going on in the world, you know what I'm saying? Which probably ain't even got to do with no drugs. It's kind of the, the stigma behind, um, your color, you know?

Slow 911 response times were linked to the belief that predominantly Black and Brown parts of the city are not prioritized when compared to predominately White areas.

“For one, I feel they don't care. For two, I feel again, they feel that we don't have an opiate problem in the black community, it is more of another race problem.”

“In any area where they live, Blacks and Brown, I don't feel that first responders ever put a priority in our areas whatsoever. Um, sometimes I've called the police, and it takes [them] forever to get to <laugh> my house. And I'm like, you know, I just can't believe that if I was in Carmel, if I was in Fishers, if I was in other areas, they would come right away.”

Exhaustion related to historic mistreatment and lack of improvement was tied to low desire to engage in interventions. Participants discussed instances where interventions were attempted or improvements were promised without being implemented effectively in their community.

“I feel like, um, Black people as a whole are exhausted. Um, and sometime when information is, um, put out like, we've been through so much that people don't even make the effort like to, um, try to get the information because they feel like ain't nothing [going to] happen or ain't nothing going to change.”

Fear and mistrust of law enforcement often involved the interplay of race-based and substance use-based stigma. Participants expressed the expectation of brutality and criminalization when police are involved following the overdose of a Black individual.

“A White person would typically be more comfortable with calling the police than a Black person would. And that's just been like that since before this was even the issue. So, um, you know, um, yeah. So, I do believe that it's more so of the fear that keeps us from calling, making that first call. You know what I mean? Cause it can go left.”

“It's a mental health issue in the Caucasian community. It's a criminal issue in the African American community.”

A few participants discussed mistrust in naloxone itself, noting lack of trust in pharmaceuticals and a legacy of race-based abuse from the medical establishment.

“I think already there's a fear with medication in general, uh, with even utilizing or even, um, using or even getting medication for anything outside of just traditional diseases like diabetes, uh, heart disease, high cholesterol. I don't think that our community would even use naloxone.”

“Most medication was developed by the White community as far back as you can think. And then it has been used in history to poison the Black community before. And so why would they trust something coming from the White community to help?”

### Intervention opportunities

#### Positive intervention experiences

While the interviews focused on perceptions of barriers, many participants discussed interventions they find helpful and ideas for reducing overdose fatalities in the future. Some descriptions of existing interventions dealt with substance use directly, while most described how other needs have been filled. Many described individuals from their community and institutions that are already trusted. Some recalled overdose interventions by substance users themselves.

“I actually had, not firsthand experience, but heard some stories of people that have been educated on it about Aaron's law and stuff and they didn't run. That's the reason they didn't. And they actually stayed there with, uh, with their friend and saved their life… So, I think educating them more and getting them more comfortable with it, doing demonstrations and really showing them testimonies of how it saves lives. I think that'll, that'll reassure people more in the community.”

“They get stuff because whoever they know don't have access to transportation. Whether it is, you know, the bike, the car or walking or whatever the case is.”

“What I know is, um, when you have people come into a community or a Black individual that comes into a community and they have experience, like with myself, I have experienced, um, drug usage and the lifestyle that come with drug usage. People are more, um, apt to, to listen to me.”

Some wide-reaching interventions that provided resources to a number of people often involved grassroots movements developed by community members themselves. Others involved institutions that had a trusted presence in the community.

“I know a place that a lot of the Black community does go to, it's called [local park]. Um, they do a lot of stuff for the community over there. Um, yeah, they do like, um, outreach events and I know that there will be a place where people will feel comfortable going to and, uh, actually engage with the people in there.”

A few participants shared their ideas about spreading information and getting naloxone to more people in their community. This included motivation to care for each other person-to-person and in small gatherings, as well as what harm reduction organizations could do to ensure people in active addiction in the study neighborhoods had easy access to naloxone and harm reduction education.

“I would probably throw a block party [because], you know, you can get everybody to come to those. And during the block party, take a minute with the music off and discuss it with the community.”

“We have to carry it more for [police] don't assume, because then they'll get tired of asking about that. You, you, you, you understand what I'm saying? It's just like when they see it more often, they can't assume everybody's on drugs that's carrying it. “But why are you carrying it?” “Cause I might can save a life today.”

“I think really, you know, that that's only [way the community is going to] be motivated and feel involved. If you constantly come into my house and I constantly see y'all out here with these shirts and out here with this stuff and if I'm constantly seeing that, it's like these people ain't going away. Well maybe they do get my best interest at hand. Let me get more involved and let me pay more attention to it.”

## Discussion

Our findings demonstrate the impact mental barriers and racism have on engagement in lifesaving behaviors following an overdose by providing narratives of the relationships between those and practical access to resources and education. Participants’ comments regarding the fear of stigmatization when carrying naloxone, especially in police interactions and mistrust of previous interventions, lend insight into why their community is not using known resources or going to places where they can acquire them. These narrations support a conceptualization of access theorized by Levesque et al. that includes acceptability, approachability, and appropriateness by showing how resource use is impacted by belief of impending maltreatment [27] as a consequence of lifesaving behaviors. Comments on lack of intervention visibility, particularly related to substance use services, indicate resources may also be lacking in pockets of the study area that are predominantly Black.

Lack of trust in and fear of institutions that provide information about lifesaving tools indicate that participants may not access naloxone resources, even when physically and financially available. Historical trends of Black communities taking low priority when resources are distributed created feelings of exhaustion and disenfranchisement. Additionally, participants shared experiences of mistrust in pharmaceutical interventions stemming from race-based mistreatment in medicine, supporting suggestions from Dayton et al. that medical mistrust has an impact on “engagement with overdose prevention services” [28].These are in line with the impact of “unmet obligations” described by Lopez et al. [29].

Race magnified mental barriers reported as reasons for hesitation of carrying naloxone and calling first responders. Participants described expectations of being searched by law enforcement due to race and fear of punitive action if naloxone is found. This supports findings regarding how double stigmatization by law enforcement [30] and racialized criminalization [29] as potential consequences of carrying naloxone and calling 911 lead to hesitancy to engage in lifesaving overdose behaviors. Responses also support fear of stigma or enacted stigma within one’s community [30] when associated with substance use by carrying naloxone. The presence of enacted stigma as well as internalized stigma identified in interview responses [31], their impact on and magnification of multiple barriers, and the related increased risk of overdose make stigma a particularly important factor to address in harm reduction activities.

While many participants detailed mental and practical barriers they have experienced or observed, most also reported willingness to learn about and carry naloxone to assist in overdose response when education and resources come from trusted community members such as Black church leaders. There was a common belief that the study community would be more receptive to naloxone education and resources if those providing information were visible in the community, especially if they were community members themselves. Some discussed favorable outlooks in fatality reduction if more boots-on-the-ground naloxone interventions were initiated. Many participants share trusted sources of assistance that involved community member-led organizations and outreach efforts. The comments on trusted help are particularly powerful as questions related to this were not included in the interview guide. These narrations came organically as part of the conversation between interviewer and participants. Indeed, there have been several interventions that were implemented on a countywide level to help opioid overdose survivors with providing naloxone and medications for opioid use disorders (MOUDS) with recovery supports [32], and law enforcement efforts that aimed at disrupting local drug markets through the seizure of opioids [33]. However, labeling these interventions and efforts as trusted sources of assistance for our participants or residents who live in these communities remains a challenge. Our participants have experienced a long history of stigmatization and mistreatment due to both race and substance use, but examples of mutual aid provide hope for improved outcomes. Responses support the benefit of mutual aid in marginalized spaces where formal services have left unmet needs [34]. Narratives of mutual aid, from people in active use or recovery from substance use, are in line with findings from Bathje et al. [35] that highlight high motivation to engage in altruistic acts among people who use substances. While history cannot be undone, the interviews provide insight into sources of trusted assistance from individuals who are experts in their own community.

As with any qualitative research study, this study has a number of limitations that are important to highlight. First, the researchers did not have any personal relationship with any of the participant interviewees; however, one facilitator of the research team, the interviewer, was part of the community under study and had lived experience related to drug abuse and subsequent successful rehabilitation. Thus, the interviewer had an *insider* and *outsider* role which allowed her to share her experience with the participants at some points of the interviews to create rapport and closeness. This prompted the participants to feel at ease during the interview. Without the interviewer's lived experience and race, it would have been difficult to find the participants that would have been willing and prepared to talk openly about their experiences. The researchers reminded the interviewer the importance of keeping an impartial position during the interviews.

## Conclusions

The knowledge shared by our participants highlights a variety of public health implications. First, lifesaving behaviors following an overdose require access to necessary resources that goes beyond their physical location. Strong perceptions of mistrust, fear, and stigmatization in relation to first responders and medical institutions help explain low motivation to use the tools they have access to, even when physically available. Interventionists should consider access to be both mental and physical, especially when engaging marginalized communities who have a history of systematic mistreatment. Second, participants’ narrations of trusted sources of care and education demonstrate the power of trust and consistent community involvement. Trusted sources of help come from community members themselves and entities that are built and/or staffed by the very people they are created to serve. Leveraging entities and individuals who already have trust in marginalized communities can surmount the barriers mistrust, fear, and stigma present. We recommend that resources be aimed at interventions driven by mutual aid frameworks and provided to the target communities as directly as possible. Third, more work should be done in exploring the intersections between substance use-related stigma and race-related stigma. While deep exploration was beyond the scope of this paper, the presence of double stigma in the interviews demonstrates that the study of intersectionality that includes substance use is necessary to increase the effectiveness of future interventions.

### Supplementary Information

**Additional file 1**: Codebook for Qualitative Analysis.

**Additional file 2**: Expanded Quotes – General Barriers.

**Additional file 3**: Intervention Opportunities.

## Data Availability

Excerpts of interview transcripts are available from the corresponding author upon reasonable request. The full transcripts generated and analyzed during the current study are not publicly available to protect personal identities.

## Abbreviation

MACRO-B: Multi-Sector and Multi-Level Community-Driven Approaches to Remove Structural Racism and Overdose Deaths in Black Indianapolis Communities
