# Supplementary material for: “That’s why we’re speaking up today”: exploring barriers to overdose fatality prevention in Indianapolis’ Black community with semi-structured interviews
Source: Harm Reduct J. 2023 Oct 27;20:159. doi: 10.1186/s12954-023-00894-8 (PMC10612233; doi:10.1186/s12954-023-00894-8)
Supplement: Supplementary file 1 — Additional file 1: Codebook for Qualitative Analysis. [file 12954_2023_894_MOESM1_ESM.docx]

**Additional File 1. Codebook for Qualitative Analysis**

| Theme | Code (Word or Phrase) | Subcode | Code Abbreviation | Detailed Description: a 1–3 Sentence Description of the Coded Datum’s Qualities or Properties |
| --- | --- | --- | --- | --- |
| *Perceptions* |  |  |  | Narrations related to beliefs and perceptions, but not direct participants’ experiences. |
|  | First Responders |  | P.FR | Institutions/organizations or people who work for community members that are the first to arrive on the scene following a crisis. This includes law enforcement, emergency medical personnel, and firefighters. |
|  |  | Community Perception of First Responders | P.FR.1 | This includes community members' perceptions of first responders' attitudes and behaviors when providing services or attending to community members. |
|  |  | First Responders' Perceptions of Community Members | P.FR.2 | This includes first responders' perceptions of community members' attitudes and behaviors when receiving attention. |
|  | Substances |  | P.SU | Illegal substances different from prescribed medications such as alcohol, cocaine, etc. |
|  |  | Use/Users | P.SU.1 | Dealing with the use of substances and/or people who use them. |
|  | Naloxone |  | P.NA | This includes community members' perception of Naloxone and/or Narcan. (e.g., many people around me believe that naloxone harms people rather than saves.) |
|  | Interventions |  | P.IN | Programs or efforts to create awareness about substance abuse and to reduce or prevent overdoses (delivered by an organization or institution). General perceptions in the community. |
|  |  |  |  |  |
| *Experiences* |  |  |  | Narrations related to a specific experience either directly observed by interviewee or a secondary account they describe. |
|  | First Responders |  | E.FR | Institutions/organizations who work for community members are the first to arrive on the scene following a crisis. This includes law enforcement, emergency medical personnel, and firefighters. |
|  |  | Interaction | E.FR.1 | This includes community members' experiences when interacting with first responders. (e.g., when policemen arrived at my house…) |
|  |  | Procedures/Protocols | E.FR.2 | This includes community members' experiences with first responders' procedures/protocols. (e.g., when I called 911, the waiting time was long. etc.) Illegal substances different from prescribed medications such as alcohol, cocaine, etc. |
|  | Substances |  | E.SU | Illegal substances different from prescribed medications such as alcohol, cocaine, etc. |
|  |  | Use/Users | E.SU.1 | Dealing with the use of substances and/or people who use them. |
|  | Naloxone |  | E.NA | Experiences related to the use of, training on the use of, and/or obtaining Naloxone or Narcan. |
|  | Interventions |  | E.IN | Programs or efforts to create awareness about substance abuse and to reduce or prevent overdoses (delivered by an organization or institution). Community members' experiences. |
|  |  |  |  |  |
| *Barriers/Threats* | Racism |  | B.RA | Narrations in which participants describe racism/racial bias that prevent people with drug addiction from receiving appropriate assistance or treatment. |
|  |  | History | B.RA.1 | Narrations in which participants discuss historical instances of racism and/or historical implications. |
|  |  | Exhaustion | B.RA.2 | Narrations in which an individual or community member describes the psychosocial stress responses from being a racially oppressed group. |
|  |  | Disenfranchisement | B.RA.3 | Narrations in which an individual or community member experiences disenfranchisement as a mean of racism. |
|  | Mistrust |  | B.MIT | Incidents in which lack of trust prevent people with drug addiction from seeking assistance from institutions, organizations, or others. Either implicit or explicit. |
|  |  | Institution/Organizations | B.MIT.1 | Narrations in which an individual or community member describes lack of confidence/trust in systems, whether they be medical, public health, and/or institution. |
|  |  | Power | B.MIT.2 | Narrations in which an individual or community members describe the lack of lawfulness and legitimacy of power, or where powerlessness is a barrier. |
|  | Fear |  | B.FE | Narrations in which participant describes emotion aroused by the detection of an imminent threat (either perceived, anticipated, or observed). |
|  | Access |  | B.ACC | Narrations that describe poor access to information and resources as a cause that prevent people with drug addiction from receiving needed care or resources. |
|  |  | Information | B.ACC.1 | Narrations where participant describes lack of information or inability to find it as a barrier to opioid overdose prevention. |
|  |  | Substance Use/ Community Resources | B.ACC.2 | Narrations where participant describes a barrier to resources. |
|  | Naloxone |  | B.NA | Participant describes barriers to anything having to do with naloxone/Narcan. |
|  | Structure |  | B.STR | Participant describes barriers related to community environment. Can include gentrification and redlining. |
|  |  | Institutions/Organizations | B.STR.1 | Descriptions of barriers related to formal organizations and institutions. This includes hospitals, first responders, policies, insurance, etc. |
|  |  | Neighborhood Location | B.STR.2 | Occurrences in which access to resources were affected by the geolocation of the person in need. |
|  |  | Infrastructure | B.STR.3 | Descriptions of barriers related to the built environment. |
|  | Stigma |  | B.STI | Descriptions of barriers related to negative views of others on an individual. |
|  | Shame |  | B.SHA | Descriptions of barriers related to negative views an individual has of themselves. |
|  | Substance use |  | B.SU | When being in active use is a barrier to getting help |
|  |  |  |  |  |
| *Strengths/Assets* | Social Support |  | S.SSU | Narrations in which the family/community/family play a crucial role to **as a resource for participant or person in need receive assistance/treat drug addiction. |
|  |  | Family | S.SSU.1 | Narrations in which an individual or community member describes the ways family members play a key role to search assistance or start a rehabilitation process. |
|  |  | Clergy | S.SSU.2 | Narrations in which an individual or community member mentions clergy as an important source of information and assistance for people with drug addiction. |
|  |  | Community | S.SSU.3 | Narrations in which an individual or community member describes how members of a community can be source of information about institutions, programs or places where people with drug addiction can find assistance. |
|  | Access |  | S.ACC | Incidents in which the access to information and resources facilitated the treatment of people with drug addiction/overdose. |
|  |  | Information | S.ACC.1 | Occurrences in which an individual or community member highlights the importance of having access to information about institutions, programs or places that assist people with drug addiction; as a differential factor for preventing and treating substances abuse. |
|  |  | Substance Use/Community Resources | S.ACC.2 | Narrations in which an individual or community member describes situations in which knowing about community resources/services can help prevent and treat substance abuse. |
|  |  | Self-Efficacy | S.ACC.3 | The believe that one is able to perform a behavior that prevents overdose and will or would if necessary. |
|  |  | Trust | S.ACC.4 | Incidents in which trust in people/institutions/organizations help people with drug addiction seek/receive assistance. |
|  | Recovery |  | S.REC | Incidents in which institutions/organizations or lived experience help people with drug addiction begin a rehabilitation process or lead them to find assistance. |
|  |  | Institution/Organizations | S.REC.1 | Narrations in which religion, rehab centers, or first responders play a critical role in drug addiction prevention and treatment. |
|  |  | Lived Experience | S.REC.2 | Narrations in which participants' lived experience plays an essential role when providing help to or building trust with people with drug addiction. |
|  | Naloxone |  | S.NA | Narrations in which knowing how to use or what Naloxone/Narcan is lead an individual or community member to give assistance to people with drug addiction or share information about the product. |
|  | Mutual Aid |  | S.MA | Occurrences in which an individual or member community practices mutual aid to prevent overdose. |
