# Supplementary material for: “That’s why we’re speaking up today”: exploring barriers to overdose fatality prevention in Indianapolis’ Black community with semi-structured interviews
Source: Harm Reduct J. 2023 Oct 27;20:159. doi: 10.1186/s12954-023-00894-8 (PMC10612233; doi:10.1186/s12954-023-00894-8)
Supplement: Supplementary file 2 — Additional file 2: Expanded Quotes – General Barriers. [file 12954_2023_894_MOESM2_ESM.docx]

**Additional File 2. Expanded Quotes – General Barriers**

| Theme | Quotation |
| --- | --- |
| Practical Barriers: Naloxone | I know in my community, um, I don't think, like on the outreach piece…like I said, I live on [local street] and I really don't see like a whole lot of people far as the drug usage piece. |
| Practical Barriers: Naloxone | I don't, I'm not sure that people do know in our community where to go and just get [naloxone]. Um, I know that you can get it, that some places you have to pay for it. But I know that, like, the health department gives it out for free. But I'm not sure everybody knows that or has access to getting to that place to get it that's on the streets. You know what I'm saying? The people who are out there in the madness. I'm not sure, if someone's not bringing it to them, that they're getting it. |
| Practical Barriers: Naloxone | **Interviewer**: That, and that kind of goes to the next question. Um, do you believe that opioid users will use more opioids if they know that they have access [to naloxone]? **Subject**: Yes. Yes. They've told me that. They basically have told me that they do, they carry it with them to protect them so that they can use. **Interviewer**: Okay. **Subject**: And then when they're hit with it and they wake back up, they just wait a little bit and do it all over again. It's that. So, so it's a little dangerous really. **Interviewer**: Mm-hmm. <affirmative>. **Subject**: Um, because they, if they're not educated about what they even have, they could really hurt themselves with the use of it, if you think about it, because not realizing how much they have in their system, they could overdose themselves multiple times because they can't feel all of the drugs in their system as it wears. You know what I'm saying? Like, it could be really dangerous and cause more overdoses. |
| Practical Barriers: Naloxone | **Interviewer**: Yes. <laugh>. Okay. And that is, um, I know you said you have the, um, injectables. **Subject**: Mm-hmm. <affirmative>. **Interviewer**: Uh, what instructions did you receive with that? **Subject**: None. None. That's why I don't even [want to] carry it [because] I have no idea how to use none of that stuff. |
| Practical Barriers: Naloxone | Um, I've never seen, like needle exchanges. They say they have them, you know. They're not in right, in the direct area where the drugs are being done in drug areas, you know, when you're using, nobody has money to get on a bus or anything. It has to be direct and centered where it's needed. |
| Practical Barriers: Naloxone | Should be. Yeah. A lot of, it's weird. A lot of people that use opiates, I'll say heroin or whatever, they have no idea on really how to even help someone recover. |
| Practical Barriers: Naloxone | **Interviewer**: And you do know where it's available in, in your area. You did say that. Now there are some legitimate claims that Black-dominated communities do not receive equitable overdose education and naloxone distribution compared to White-dominant communities. Do you agree or disagree? **Subject**: I agree. I mean, because now that you say that, I don't see it advertised anywhere. Now, I know that you could get it down at this health clinic. **Interviewer**: Mm-hmm. <affirmative>. **Subject**: With a person that's, um, homeless or just didn't know. I don't see it advertised anywhere. **Interviewer**: Okay. **Subject**: I don't see any advertisements for that. “We have naloxone free here.” I never see that, really. |
| Practical Barriers: Naloxone | Then within two weeks he was dead at his aunt's house around the corner, which is still in 46218. Um, [because] they didn't have any idea what to do. |
| Practical Barriers: Naloxone | Narcan training should be implemented at different places. So, then people could be trained to know what to do. Like when I left [treatment center], they gave me like four Narcan kits. I didn't even know what it was, but I figured it is a nasal and I read it and then coming here doing the volunteer work, I figured it out. So, [there] should be more education and training on what it is. |
| Practical Barriers: Naloxone | **Interviewer**: So, you never encountered either the naloxone or the training or anything in your community? **Subject**: No. Took just past couple weeks ago and [B] came and gave our house a training. That was it really. That was the first time. Never knew anything about it. |
| Practical Barriers: Naloxone | **Subject**: No. No. No. I never heard of Narcan kits until I went to rehab. **Interviewer**: Okay. **Subject**: Never ever, like Narcan, I thought I knew about the, the pin thing. Maybe whatever that is. Like when you are [going to] have an allergic reaction. **Interviewer**: Oh, EpiPen. **Subject**: EpiPen. **Interviewer**: Mm-hmm. <affirmative>. **Subject**: But I've never heard of Narcan until I got in, went to rehab. And that was six, not even three months, four months ago. |
| Practical Barriers: Naloxone | **Subject**: I did not know this place existed until today. **Interviewer**: Really? **Subject**: Until [G] asked me to participate in this program. And I got a call from your admin and I got the meeting set up for the address. I did not know that this was the place for this type of thing. And I lived in 46208 most of my life. |
| Practical Barriers: Naloxone | I think the reason [naloxone] is in certain areas is because it's more opioid addiction in those areas. **Interviewer**: Okay. **Subject**: I, yeah, I won't put it on the race thing. I want, I just [want to] say that some areas is more infested with cocaine and crack cocaine and more areas are infested with heroin and opioids. So, I think that's why they reach those areas more so than they do the areas that the overdoses [are] limit…you know what I'm saying [are] lower. |
| Practical Barriers: Naloxone | **Interviewer:** Where they can go to get the naloxone, are people aware of where to go? **Subject**: I would say not enough. I, I would say probably one fourth if we're putting it in…a fourth. I would say one fourth of the community knows. |
| Practical Barriers: Naloxone | I don't think it's enough and the people that do get it, maybe they just don't, you know, don't spread the word. But I like, it's just small pockets, you know, it's just really small pockets. And then there's some people that just had no idea of, of that this stuff was even available anywhere in the community. So, um, from my perspective, I think it's more about getting the, getting the education and word out there about it. |
| Practical Barriers: Naloxone | **Subject:** If we didn't know nothing about, uh, Narcan or, you know…**Interviewer**: You didn't know where they, you could go in your community to find it or…**Subject**: Nowhere in the community to find it. Because when I got high, I got high all over the city. So, you can be in one zip code or this <inaudible> or this zip code. |
| Practical Barriers: Naloxone | Even though I…[naloxone] was out there, I didn't even know about that stuff. People had OD'd in my areas when I was in my addiction. Only thing we knew was to put [them] in the tub and run the cold water on. |
| Practical Barriers: First Responders | But other than that, I'm running from you, the policeman. I'm, I'm gone. I don't know about no law. You don't even know nothing about the law. IPD you need to be educated about Aaron's law because your initial thing is to arrest. |
| Practical Barriers: First Responders | They don't. They, and I don't think when they get arrested, like they don't know how to speak up for [themselves]. Or even the public defenders, they’re not educated about the services to me. So, if they’re not educated, people just sit there and [get] locked up. |
|  |  |
| Mental Barriers: Stigma | **Interviewer**: Do you think that race is a factor into why they don't call or they just in general, people don't call? **Subject**: I believe, well, I know it's because of the stigma. Um, the way society look at people, um, they use drugs and drugs ain't got no color. <laugh>. **Interviewer**: Okay. **Subject**: Drugs do not have a color. Um, it affects all races. So, I believe, you know, um, it is because of, like I said, the stigma, um, them being possibly mistreated. You know? Whether it be by police or it is, um, by individuals because people really don't have a, a knowledge or understanding. |
| Mental Barriers: Stigma | I've seen EMS treat people horribly that are inter, that are, are users. I've seen them call them junkies to their face. Like I've seen EMS behave horribly. Like, like they have no type of stigma education whatsoever. |
| Mental Barriers: Stigma | **Interviewer:** What do you think, um, do you know of any structural barriers, community conditions, policies, practice, misinformation, stigmatizing beliefs that contribute to inequitable overdose education and naloxone distribution in the Black communities? What is standing in our way? **Subject**: I hate to say we are. **Interviewer**: Okay. You want to expand on that? **Subject**: I, I think that's, you know, I think, um, I don't, I don't know. Maybe the younger generation is more open and, you know, um…But I think that the older generation, like our older people, like my age and older, um, aren't so open and accepting and forgiving and understanding. They don't seem to be. I don't think that…they just, you know, “you should just stop it. Just put it down.” You know? I don't know that they've been educated enough. |
| Mental Barriers: Stigma | If they find [naloxone] on them, like they assume like, “Oh, if you got this then you may have some drugs somewhere. Let's go ahead and strip search her, or strip search him and see where it's hidden.” Or you know, like they wouldn't understand that it's a per, it is a preventative something. |
| Mental Barriers: Stigma | Because of the [stigma] that's put on drug use. Um, people are afraid that, because they carry it, you know, they're [going to] be affiliated. |
| Mental Barriers: Stigma | **Subject**: People associate overdoses with like, shooting up or anything. You can, somebody can just try to commit suicide, take a whole bunch of pills. **Interviewer**: Right. **Subject**: Or so, so they’re not educated when they are thinking like, its meth and heroin and stuff like that. |
| Mental Barriers: Mistrust | They just rather just get away from where [an overdose is] going on so that they don't have to be a part of the process of the police…dealing with the police. [Because] they don't like dealing with the police. |
| Mental Barriers: Mistrust | **Interviewer:** Due to the association between carrying naloxone and opioid and other drug use, do you think that people are reluctant to carry it if they might be harassed by police if they do? **Subject**: Um, yeah. I would say there's a hesitance because of the trust factor in, in our community. You know what I mean? We don't trust the police as it is already. So, um, I know from my experience working a little bit, uh, besides [harm reduction org], when I was, uh, an employee at [social service], we would do a lot of outreach, stuff like that. And people were just not, they were scared because they, like, they didn't want to go to jail. They’re like, “We don't know nothing about this stuff.” I'm not going to, they are thinking like it's something wrong with it. Like, yeah, like “If the police catch me with this, maybe this will be an indication that I'm doing something.” It was more a hesitance like that. |
| Mental Barriers: Mistrust | You know, but then you got some that just, you know, you don't want to fool with. They strictly, just strictly in their mind that this is how it's [going to] be. I ain't giving you a chance. And you know, so that, that makes it hard for people to call the law because you don't know what, what type of person you're [going to] get. |
| Mental Barriers: Mistrust | I think they're reluctant to call because I think they think [they’re going to] get in trouble. |
| Mental Barriers: Fear | Some people do have reservations, you know, in wanting to help because, uh, they may be afraid that if something goes awry, that they may be sued. You know? Not knowing fully that, that there's a, a Good Samaritan law that protects that. |
| Mental Barriers: Fear | I feel like people are scared when they're all participating in, in activities they shouldn't be. So, they don't want to call the people for help. So that's a big deal that I'm hearing now. Like, instead of calling for help, they'll put a person outside...I do believe it's because people are intoxicated [themselves] at the moment. They're afraid of repercussions themselves. |
| Mental Barriers: Fear | I think the older generation would fear carrying it. You know? Because they just don't know. They just don't know what to do and they don't want to get involved. |
| Mental Barriers: Fear | I think already there's a fear with medication in general, uh, with even utilizing or even, um, using or even getting medication for anything outside of just traditional diseases like diabetes, uh, heart disease, high cholesterol. I don't think that our community would even use naloxone. So, I definitely don't think that most people would carry it. |
| Mental Barriers: Fear | I know one reason that I particularly wouldn't [want to] carry it, there would be a fear of being responsible for whether it worked or whether it didn't work. Uh, I don't [want to] be responsible for someone else dying. Um, and also the idea, I think heavily in our community, there's, there's, um, there is, uh, other avenues that people will take in order to stop using before they'll take naloxone when someone's in overdose. |
| Mental Barriers: Fear | When they're in that, in that emergency situation, when that person is in the emergency situation, I don't think that many people want to call an ambulance or a crisis intervention team because they may get law enforcement instead. I said so, so that call already, you know, you're calling a gun to somebody, you know, you're calling a gun to someone who's actively overdosing, as opposed to an ambulance showing up and actually being able to give them the medication that they need in order to revive [them]. |
| Mental Barriers: Fear | **Subject**: I wouldn't go to a fire station because they're just like the police, but I would assume the fire stations would have [naloxone] as well, but I wouldn't go to the fire station. **Interviewer**: Okay. **Subject**: Or to the police station. |
| Mental Barriers: Fear | **Interviewer**: Okay. So that answers the next question. Um, are you willing to carry naloxone with you in case you encounter an overdose victim? **Subject**: Um, probably not. And probably because of the aforementioned question before about law enforcement. Like, I just don't want anything extra in my car. |
| Mental Barriers: Fear | I just wouldn't want the personal responsibility of having that on my person. |
| Mental Barriers: Fear | I could totally understand why other people would be fearful to carry it because, you know, well if you've got it, then you must be using. You know what I mean? |
| Mental Barriers: Fear | Um, I think some people might be afraid of, uh, what might happen when they do, when they use it. Um, [because] I know, like when I became CPR certified, we have to ask them like, “Hey, can I give you CPR?” Which is a little weird because like, what if they can't talk? You know? But, um, I understand consent. Um, but yeah, I think some people would be worried about consent, probably. They'd be like, oh, like, I don't [want to] get into legal trouble if I do this for this person. |
| Mental Barriers: Fear | It's one of those topics that a lot of people are scared to discuss. They feel uncomfortable with discussing this because people get the wrong ideas. “Hey, I might try to discuss Narcan with a friend and they might think I do drugs.” |
| Mental Barriers: Fear | Some people are afraid to carry it because if they administer it and they do something wrong with administering it, they'll go to jail. So, some are okay with it, some aren't because most people that are in drug addiction won't think like that, so that's why they don't carry it. |
| Mental Barriers: Fear | You know, you can be with people that know, know about it, know you carry it. But will they take time out to help you? You know, a lot of people again, I say, run from that. |
| Mental Barriers: Fear | I don't think it's a race thing. Mm-hmm. To some of, to some degree. I don't believe it is. I mean, I just think that it's a thing of the fear factor in it. You know, rather it be White or Black. I think all drug addicts have a fear factor of dealing with the police. It's not, I don't think it's really a Black or a White thing. I think it's just a drug addiction thing. |
| Mental Barriers: Fear | So many of [them] are afraid to use it because they're afraid that, “Okay, I do this and they don't come out of it maybe and then they end up dying anyway. Maybe I did something wrong. Maybe I was the one that killed them.” |
| Mental Barriers: Fear | **Interviewer**: Do you, um, some people feel afraid to call the EMS or call the police and…**Subject**: Yes. **Interviewer**: Why do you think that is? **Subject**: Because even though you tell them that they won't get in trouble, “Just call,” they very feel that that's not so because of the law. You know? Because they know they’re doing something they ain't got no business doing. And they know that, you know, when [police] get there, they nine times outta 10, [are going to] want the instrument…what if it's some dope still left? [The police are going to] want that. And so, if [the instrument and dope still there,] then they think it’s probable cause. They [will] probably get arrested. |
| Mental Barriers: Fear | Yeah, that is a fear. Even they, they have a fear of carrying clean syringes because they like, they get like pulled over, like they going to jail. Or, say I don't do drugs. So, say if I got [naloxone] in my car, then [police are going to] associate me, you know, doing drugs or something and I [have to] take all these classes even though I don't do drugs. |
