# Supplementary material for: “That’s why we’re speaking up today”: exploring barriers to overdose fatality prevention in Indianapolis’ Black community with semi-structured interviews
Source: Harm Reduct J. 2023 Oct 27;20:159. doi: 10.1186/s12954-023-00894-8 (PMC10612233; doi:10.1186/s12954-023-00894-8)
Supplement: Supplementary file 3 — Additional file 3: Intervention Opportunities. [file 12954_2023_894_MOESM3_ESM.docx]

**Additional File 3. Intervention Opportunities**

| Theme | Quotation |
| --- | --- |
| Witnessed | Passing stuff out at places like going to the YMCAs and the Boy’s Clubs and all that. You know, stuff like downtown passing stuff. A route passing stuff. [Because] there's plenty of people. Like I said, I was downtown getting lines for free food. Put that, have somebody downtown giving that stuff out. |
| Witnessed | I, when you talk about my community, I don't think so much as far as my, in my immediate community because my children were raised in addiction. So, my children knows that it works. My daughter has naloxone in her car now because she's had to use it for other people. So, my immediate community, because of who I am, and they've been open to what I do, um, are not, they're, they're fine with it. |
| Witnessed | **Subject**: And I think the, like I said, the only reason why we had it was because of my ex-husband. But my daughter has, has used naloxone so many times on her boyfriend. Like she has had to use it for, but her friends and people, [because] she's that age group. |
| Witnessed | **Subject**: I feel like a lot of times in a lot of situations they need somebody to be an accountability partner. Um, somebody that's, that's been there that understands and can say, you know what, “Hey you got this man,” or “You got this girl, you good. You all right? You can do this.” **Interviewer**: Mm-hmm <affirmative>, **Subject**: “When it gets rough, call me.” |
| Witnessed | Just because, like I said, you never know where. Because I know the gas stations, a lot of addicts hang out around gas stations and stuff like that. So, it probably would be wise for me to maybe carry it, considering I have a little education on it, about it, and what it can do to help someone. |
| Witnessed | I actually had, not firsthand experience, but heard some stories of people that have been educated on it about Aaron's law and stuff and they didn't run. That's the reason they didn't. And they actually stayed there with, uh, with [their] friend and saved [their] life, you know, as opposed to other times they said they done. All they did was run and didn't look back to know, you know, to even figure out what was going on. So, I think educating them more and getting them more comfortable with it, doing demonstrations and really showing them testimonies of how it saves lives. I think that'll, that'll reassure people more in the community. |
| Witnessed | It was help out there in the community, everybody. You know? It was just always, luckily somebody just knew…had experienced [with] somebody going out, you know, ODing and bringing them back, you know, by using the, the old method. You know? Or somebody happened to know it was a nurse, “Go run, get the nurse in the apartment so and so,” and you know, she told [them] what to do. You know, it was, that's the only thing we knew. |
| Witnessed | They get stuff because whoever they know don't have access to transportation. Whether it is, you know, the bike, the car or walking or whatever the case is. So, you know, they help each other. |
| Witnessed | What I know is, um, when you have people come into a community or a Black individual that comes into a community and they have experience, like with myself, I have experienced, um, drug usage and the lifestyle that come with drug usage. People are more, um, apt to, to listen to me. |
| Witnessed | And the Black people don't have like a whole lot of trust. So, they were more apt to, to trust me and listen to me versus listening to y'all. And then a lot of times, with a Black community, you got the poverty and you have people that might not have a high school diploma or might not have the best kind of job or anything like that, they’re more apt to listen to somebody that's just like them. |
| Witnessed | It was a guy that came in at, um, um, a pizza place. And he came in and he wanted some [naloxone] because wherever this pizza place is must have a whole lot of like, homeless and people of that nature. So, um, he wanted to get some, he wanted to learn how to administer it. Um, and he had, I guess he had some instances where, you know, people didn't pass out, you know what I'm saying? And so, he wanted to, to know more about it and how to administer it and you know, how to, what he could do to help. |
| Witnessed | I was trained in naloxone use by the [local harm reduction charity], um, six years ago. So since then, I just kind of been like keeping it on me and trying to teach whoever wants to learn about it. |
| Witnessed | **Interviewer:** You spoke of [local harm reduction charity]. Are they at one location or…**Subject**: Um, she, she's the person. You call her, she'll pull up. **Interviewer**: Okay. **Subject**: Right to your house, drop it off to your house. Um, she'll call you and be like, “Hey, are you low? You running out?” So she's more of a feet-to-the-ground type person. You know what I mean? She doesn't actually have…specifically have an office at the moment. |
| Witnessed | I know a place that a lot of the Black community does go to. It's called [local park]. Um, they, he does a lot of stuff for the community over there. Um, yeah, they do like, um, outreach events and I know that there will be a place where people will feel comfortable going to and, uh, actually engaging with the people in there. |
| Witnessed | **Subject**: There's a guy named [J] that works in [local park] and he’ll be, yeah, he'll be more than welcome to talk and help out. **Interviewer**: Okay. Do, are people aware that they can get services from [J]? **Subject**: Mm-hmm. <affirmative>. **Interviewer**: Okay. **Subject**: The community know [J]. He, he talks to his people. |
| Witnessed | **Interviewer**: And you, you spoke of, uh, the box, um, over at [local pastor’s]. **Subject**: [Local church], in our church. Yes. |
| Witnessed | I couldn't get high, I couldn't drink. I did it sober. And I wouldn't have been able to if it had not have been for the support of the women in [local recovery group]. The people around me, I, I think you might know my sponsor, [L]. And so she's…look at God, girl. I know, I know...Thank God for [L]. Okay? Because I got sober at Cocaine Anonymous and thank God, Cocaine Anonymous used the big book of Alcoholics Anonymous. Which is why, and my home group's an AA meeting. |
| Witnessed | So when we called, finally called the paramedics, they were like, “Just be honest, he won't get in trouble. Tell us what he took, what did he do so we can [administer it to] him and try to save his life.” Well, they tried and they…it was just too late. |
| Witnessed | We reached out to my, uh, oldest sister's husband, um, who is now deceased, Pastor [M]. He worked at [a local church]. Um, and so that pastor told us what to do. |
| Witnessed | And so now he's grown. He's 22. His friends are starting to use and he’ll be like, “Man, y'all don't need to do that.” You know? “Y'all don't need to be trying this,” you know, “This and this and that.” And he talked to a young lady that she was using. He said, “Look, don't call me and tell me cash app you some money so you can get some weed. Cuz I ain't with all that.” |
| Hoped For | I think more people probably would carry [naloxone] if it was more accessible in, in a, in my area. |
| Hoped For | I think that that information…providing that information as opposed to only, um, naloxone only, uh, education about naloxone and opioid abuse…providing this information in relationship to all of this, I think that that would honestly be, uh, more palatable to Black people. |
| Hoped For | But we have to carry [naloxone] more [so police] don't assume. Because then they'll get tired of asking about that. You, you, you, you understand what I'm saying? It's just like when they see it more often, they can't assume everybody's on drugs that's carrying it. But “Why are you carrying it?” “Cause I might can save a life today.” |
| Hoped For | **Subject**: They need to be at the gas stations. You know what I mean? Like, because the places where we hung out, you know what I mean? My people, we know, right? Like, I always tell everybody. I said, you know, I call what we have, we call like…I call it “Dope-dar.” And the reason why I call it “Dope-dar” or “Alcohol-dar” is because we see each other like, I could spot me. You know what I mean? In active addiction. |
| Hoped For | Narcan training should be implemented at different places. So, then people could be trained to know what to do. Like when I left [treatment center], they gave me like four Narcan kits. I didn't even know what it was, but I figured it is nasal and I read it. And then, coming here doing the volunteer work, I figured it out. So, it should be more education and training on what it is so people could know. |
| Hoped For | **Subject**: So, like I said, it needs to be more, like…They have, um, the signs on the buses, inside the buses. It should be something like, up there. Because people who are on the bus, they read those things and they get information from, you know, those things. And you know, like the little things that y'all have with the numbers on it. **Interviewer**: Mm-hmm. <affirmative>. **Subject**: It should be put up somewhere. Signs and plaques. Or whatever you want. Like, **Interviewer**: Yeah. **Subject**: markers in front of the, the bus stops or whatever. Like…**Interviewer**: Absolutely. **Subject**: You know, just…**Interviewer**: Absolutely. **Subject**: Something more out there. Because I'm 49 and I never knew anything about that. |
| Hoped For | **Interviewer**: You said they don't have the education about it. Do you think it's available to them to get education? **Subject**: I think it's some, there are places that will educate you about it. But again, I think if they had like more outreach programs in the facility, in the neighborhoods that might…or would go around to locations that they know is heavily drug infested or whatever, and pass out literature. You know? Whether they read it or whether they just toss it to the side, they know the outreach is there. |
| Hoped For | So, if people would, and again, it goes back to us being connected and educating others. Um, I think that if we had people that are willing to come down to provide that education to people who are actively in addiction or who have overcome, um, I think it would, it would help. It would help. |
| Hoped For | But I do know it's a, a church that has, and that would be a good place. It's a, it's a church that has, um, they give out free food. |
| Hoped For | **Subject:** Cause once that one…like it only takes that one person out the group to get educated and then they'll go back and tell their, you know, people, “Oh, I wanna do that training too.” And so it's just…it only takes one. <laugh> |
| Hoped For | **Subject**: Right. Like I see when you go to different…I'm going to go back to like the liquor store. You can go in the liquor store, and they'll have a flyer for lawn mowing care or food. Somebody's selling food. But nothing about [harm reduction agency]. **Interviewer**: Okay. **Subject**: Nothing about this. Stores have, they have, you can, you know, put a little, you know, maybe a sign on the door. **Interviewer**: Mm-hmm. <affirmative>. **Subject**: About or you know, and like even have the little number at the bottom where they can tear it off. **Interviewer**: Right. **Subject**: Stuff like that. Like just put it out there. Put it like in the community that there are places to get help. [Because] I'm, I lived on [cross streets] and never knew that this was right down the street. You know, and like [cross streets] is a high drug area. |
| Hoped For | **Interviewer:** How do we get that done? **Subject**: Um, more than just the case workers. Word of mouth. Marketing, Facebook, Instagram, there’s so many addicts that will get on social media every day that know they need help. People like me, my friends, my family, putting a word out on the street, the churches… |
| Hoped For | **Subject**: I would probably throw a block party [because], you know, you can get everybody to come to those. And during the block party, take a minute with the music off and discuss it with the community. |
| Hoped For | **Interviewer**: With education and, and naloxone distribution, do you know, have any ideas? **Subject**: Um, I think more, yeah, more intimate. Like a door to door, corner to corner, block to block presence of it. Cause people are going to be out in the communities. But if I don't see you out, I ain't gonna know what's going on. **Interviewer**: Mm-hmm. <affirmative>. **Subject**: So, I'm thinking more intentional, like intimate, uh, door to door knocking education. Maybe picking a street to, to promote on one time block by block. Um, really promoting like town halls and stuff. The education around it. |
| Hoped For | I think really, you know, that that's only [way the community is going to] be motivated and feel involved. If you constantly come into my house and I constantly see y'all out here with these shirts and out here with this stuff and if I'm constantly seeing that, it's like these people ain't going away. Well maybe they do got my best interest at hand. Let me get more involved and let me pay more attention to it. |
| Hoped For | Just in case, like if I ride the bus and I see someone overdosing and I had it on me, I would be able to help instead of just sitting there looking and waiting around for the police to come. |
